# Recent Advancements in High-Frequency Ultrasound Applications from Imaging to Microbeam Stimulation

**DOI:** 10.3390/s24196471

**Published:** 2024-10-08

**Authors:** Min Gon Kim, Changhan Yoon, Hae Gyun Lim

**Affiliations:** 1 Department of Biomedical Engineering, University of Southern California, Los Angeles, CA 90007, USA; 2 Department of Biomedical Engineering, Inje University, Gimhae 50834, Republic of Korea; 3 Department of Biomedical Engineering, Pukyong National University, Busan 48547, Republic of Korea; hglim@pknu.ac.kr

**Keywords:** high-frequency ultrasound imaging, high-frequency ultrasound microbeam, machine learning with high-frequency ultrasonic signals

## Abstract

Ultrasound is a versatile and well-established technique using sound waves with frequencies higher than the upper limit of human hearing. Typically, therapeutic and diagnosis ultrasound operate in the frequency range of 500 kHz to 15 MHz with greater depth of penetration into the body. However, to achieve improved spatial resolution, high-frequency ultrasound (>15 MHz) was recently introduced and has shown promise in various fields such as high-resolution imaging for the morphological features of the eye and skin as well as small animal imaging for drug and gene therapy. In addition, high-frequency ultrasound microbeam stimulation has been demonstrated to manipulate single cells or microparticles for the elucidation of physical and functional characteristics of cells with minimal effect on normal cell physiology and activity. Furthermore, integrating machine learning with high-frequency ultrasound enhances diagnostics, including cell classification, cell deformability estimation, and the diagnosis of diabetes and dysnatremia using convolutional neural networks (CNNs). In this paper, current efforts in the use of high-frequency ultrasound from imaging to stimulation as well as the integration of deep learning are reviewed, and potential biomedical and cellular applications are discussed.

## 1. Introduction

Significant progress has been made utilizing high-frequency ultrasound with significant improvements in spatial resolution, though with a trade-off in penetration depth [1,2]. To boost spatial resolution, earlier research has increased the bandwidth, the f-number (the ratio of the focal distance to the spatial dimension of the transducer), and the operating frequency of the ultrasound transducer, as axial and lateral resolutions are proportional to the pulse bandwidth, the f-number, and the wavelength [1,2]. With improved resolution down to tens of micrometers, this article provides an overview of various biomedical applications using high-frequency ultrasound. First, the development of high-frequency ultrasound imaging systems, which offer enhanced resolution for more detailed visualizations of small or superficial structures, is introduced, along with its applications. Furthermore, cellular applications have been investigated, including the use of acoustic tweezers, stimulation, transfection, and the measurement of acoustic radiation and trapping forces. Additionally, the integration of machine learning with high-frequency ultrasound represents a transformative approach in the biomedical field, particularly in the realms of cancer and blood cell analysis. High-frequency ultrasound offers high-resolution capabilities, which, when combined with advanced machine learning techniques, can significantly enhance diagnostic precision and efficiency. This review explores the confluence of these technologies, detailing their applications in cancer cell and blood cell analysis.

## 2. High-Frequency Ultrasound Imaging

Most high-frequency ultrasound imaging systems developed in laboratories use a mechanical scanning system with single-element transducers since it can be implemented in a simple and cost-effective manner [3,4,5,6,7,8,9,10,11]. The imaging systems typically consists of a micro-positioning stage with a linear motor or wobbler to provide two-dimensional images using a scanning high-frequency ultrasound transducer over the field of view, along with data acquisition hardware. Signal and image processing are performed on either a field-programmable gate array (FPGA) or a personal computer (PC). From the perspective of imaging systems, the main difference between high-frequency and conventional ultrasound imaging systems is the operating frequency, which is approximately ten times higher in high-frequency ultrasound imaging systems. Thus, a high-precision mechanical scanning system (<10 μm) is required. Other than that, signal and image processing are nearly identical to those used in conventional ultrasound imaging systems.

Following the success of these prototype imaging systems and their pre-clinical and clinical applications, high-frequency ultrasound imaging systems known as micro-ultrasound were commercialized, including a pre-clinical system (Vevo 770, FUJIFILM VisualSonics Inc., Toronto, ON, Canada) and clinical system (Dermascan, Cortex Technologies, Aalborg, Denmark). Although single-element systems have performed well in both the research field and the market, they have significant drawbacks such as a low signal-to-noise ratio (SNR), low frame rate, and fixed focusing capability. In addition, their ability to provide real-time functional information (Doppler blood flow) is limited.

To overcome the problems with mechanical scanners, high-frequency ultrasound imaging systems using array transducers, in which images are formed by electrical scanning, have been developed [12,13,14,15]. In 2009, commercial high-frequency ultrasound transducers with center frequencies ranging from 15 to 50 MHz were launched by Visualsonics, along with an imaging system. High-frequency ultrasound imaging systems based on array transducers employ a digital dynamic receive beamformer, which is the most important component in ultrasound machines since it determines the final image quality, i.e., a good SNR, contrast, and lateral resolution. In a digital receive beamformer, the backscattered echo data from each element are dynamically summed by compensating for their different arrival times. For this, the received radio frequency (RF) data need to be sampled at a rate of four times the center frequency (4*f*_0_, where *f*_0_ is the center frequency). In addition, four-fold interpolation is typically applied to increase the time-delay resolution (up to 16*f_0_*). Thus, high-frequency ultrasound imaging systems require more complex analog and digital circuits than clinical systems, leading to higher costs and increased implementation challenges.

Several beamformer architectures for high-frequency ultrasound imaging systems have been proposed. Hu et al. used a four-tap fractional delay filter as an interpolator filter to minimize computational complexity [16]. In addition, they further reduced the complexity by half using the symmetric property of a linear array transducer. Yoon et al. presented a post-filtering beamformer architecture, in which channel data with the same fractional delay (i.e., 16*f*_0_ resolution) are summed prior to interpolation [17]. Thus, it can be implemented using only four polyphase filters. As an interpolation filter, a quadrature bandpass filter (QBPF) was utilized, allowing decimation during beamforming. Although these methods can reduce the computational complexity of beamformers, the backscattered RF data are sampled at 4*f*_0_, thus high-speed analog circuits (e.g., analog–digital convertors) are still required. More recently, a sub-Nyquist sampling (or bandpass sampling) technique was adopted to reduce the sampling frequency [18]. In the method, the sampling frequency could be reduced by a factor of 3 (i.e., 4/3*f*_0_) with an assumption that the fractional bandwidth of RF data is less than 67%. This is valid for high-frequency ultrasound transducers since the fractional bandwidth is not as wide as that of a clinical one [19]. In addition, they also used the post-filtering architecture; thus, both the complexity of analog and digital circuits could be minimized.

**Figure 1:**
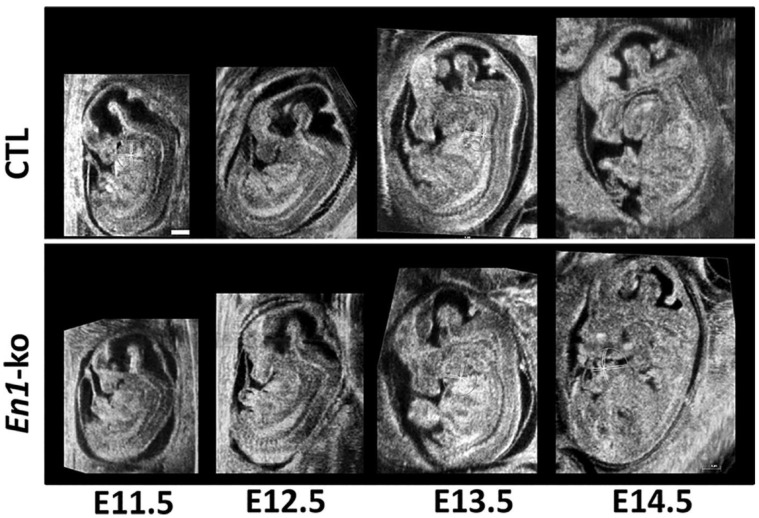
In utero longitudinal high-frequency ultrasound images of control and *En*1-ko embryos over E11.5 and E14.5. E stands for embryonic day. Scale bar = 1 mm. Reprinted with permission from [25].

**Figure 2:**
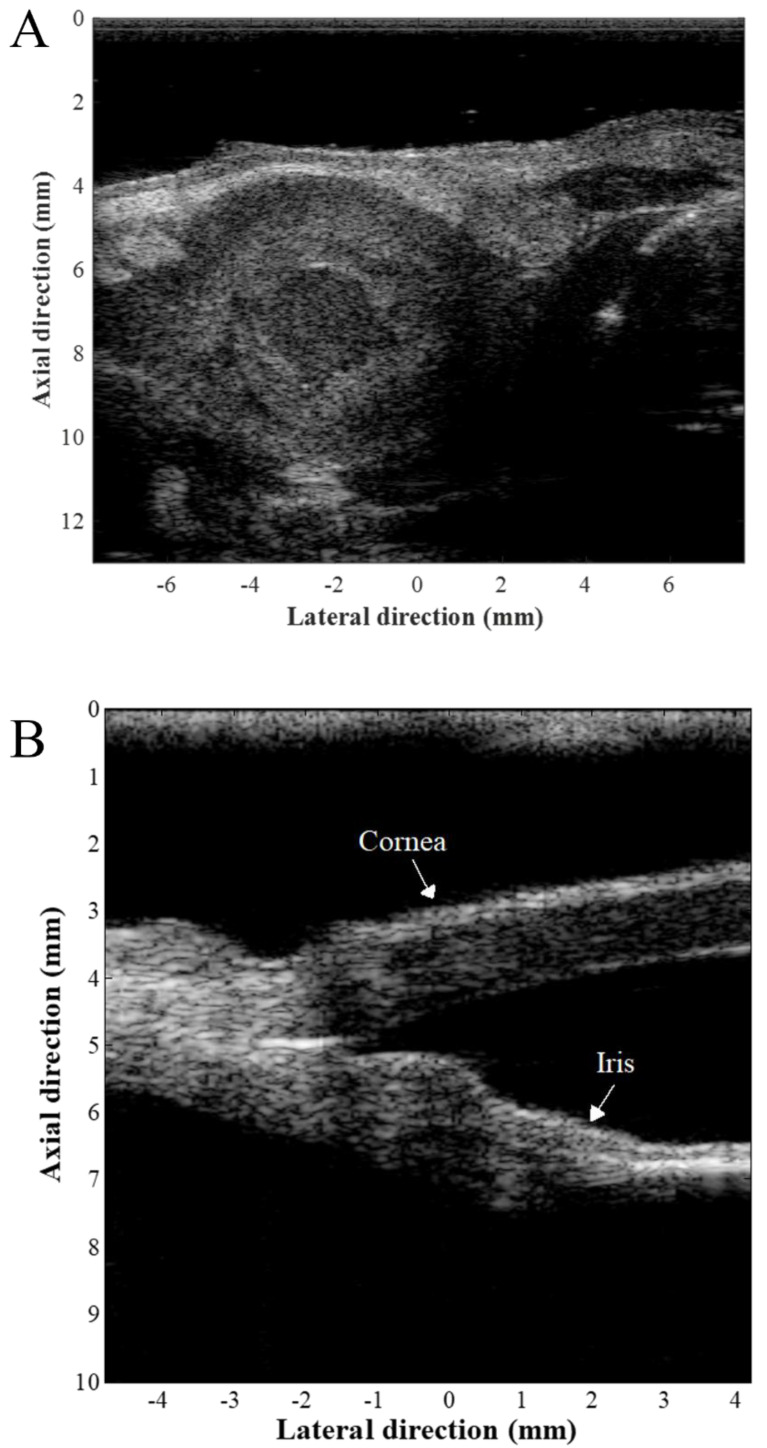
High–frequency ultrasound images of (**A**) a tumor–bearing mouse model and (**B**) an eye excised using a 30 MHz linear array. Reprinted with permission from [17,18].

Many pre-clinical and clinical applications have been investigated, such as through cardiovascular research, in cancer, in developmental biology, and on skin and ocular imaging [20,21,22,23,24,25]. Figure 1 and Figure 2 show examples of high-frequency ultrasound imaging. *Engrailed*-1 knockout (*En*1-ko) mutant mouse embryos and control (CLT) littermates were scanned using a 40 MHz annular array. As shown in Figure 1, high-frequency ultrasound imaging could visualize the morphological phenotypes in the developing brains, limbs, and heads of the *En1*-ko embryos. In the study, it was demonstrated that the mid-hindbrain deletion, which is an obvious phenotype in *En1*-ko embryos, was successfully detected at each developmental stage with 100% reproducibility. Figure 2 shows an in vivo murine mouse model with breast cancer and ex vivo eye images excised using a 30 MHz linear array transducer [17,18]. Due to its ability to provide high-resolution images, high-frequency ultrasound imaging can visualize fine details of anatomy with sub-millimeter spatial resolution. In summary, high-frequency ultrasound imaging systems will become more widely available with affordable prices and will expand their pre-clinical and clinical applications.

## 3. High-Frequency Ultrasound Microbeam

### 3.1. Cellular Application I: Acoustic Tweezers

Acoustic tweezers using high-frequency ultrasound microbeam have shown the capability to non-invasively manipulate single cells or micron-sized particles using either a single-element transducer or an array transducer. Theoretical studies by Lee et al. [26,27], utilizing a ray acoustics approach, demonstrated that a highly focused high-frequency Gaussian beam can facilitate the acoustic trapping and manipulation of spherical objects at various positions. Their results suggest that an object can be attracted and trapped by a tightly focused ultrasound beam when the acoustic gradient force from refraction surpasses the scattering force from reflection. These theoretical predictions were experimentally confirmed using a single focused Gaussian ultrasound beam. Subsequent studies by Lee et al. [28,29] demonstrated the capability of trapping individual lipid droplets with diameters of 125 µm and leukemic cells averaging 10 µm in diameter using high-frequency single-element focused transducers. A 30 MHz transducer made of lithium niobate (LiNbO_3_) was employed for lipid droplets, while a 200 MHz transducer composed of zinc oxide (ZnO) was used for leukemic cells. Expanding on the experimental work that established the viability of single-beam acoustic tweezers using Gaussian beams, Azarpeyvand et al. [30] conducted a mathematical analysis revealing the potential for manipulating both spherically and irregularly shaped Rayleigh particles with varying mechanical properties. Lam et al. [31] further developed both 200 MHz lensless LiNbO_3_ and ZnO transducers, demonstrating that single microspheres with diameters of 5 or 10 µm could be effectively trapped by these microbeam devices over distances of tens of micrometers in distilled water. Additionally, investigation on the elastic properties of breast cancer cells have been conducted using a 193 MHz LiNbO_3_ focused transducer [32]. In the study, a 5 μm fibronectin-coated polystyrene microbead attached to an MCF-7 cell was drawn toward the focus to induce mechanical deformation of the cell membrane, and thus, they assessed the relationship between the membrane’s stretched length and the trapping strength by adjusting the excitation voltage amplitude applied to the transducer. Lam et al. [33] demonstrated the feasibility of manipulating non-spherical objects, such as single red blood cells and fertilized zebrafish eggs with diameters of 1.6 mm, using a 60 MHz LiNbO_3_ transducer. Meanwhile, a transducer with a significantly higher operating frequency has also been reported [34]. A 394 MHz LiNbO_3_ needle-type ultrasonic transducer was developed that was capable of selectively controlling microparticles ranging from 3 to 100 μm in size by adjusting the excitation frequency. In addition, Lim et al. [35] developed and employed a 410 MHz LiNbO_3_ focused transducer to trap and separate red blood cells (RBCs) from RBC aggregates with a force of 391 pN. This is significant as it was the direct measurement of the inter-RBC force in the range of hundred pNs with a high-frequency ultrasound transducer. In a subsequent study, Lim et al. [36] investigated the increased deformability of highly invasive cancer cells, providing measurements of Young’s moduli for various cell types: 1.53 kPa for MDA-MB-231 cells (highly invasive breast cancer cells), 2.65 kPa for MCF-7 cells (weakly invasive breast cancer cells), and 2.77 kPa for SKBR-3 cells (also weakly invasive breast cancer cells). In addition to using a highly focused single-element transducer, a 64-element, 26 MHz phased array with electronic scanning was used to accurately trap and move 45 µm diameter polystyrene microspheres [37]. Furthermore, Yoon et al. [38] showed that a single-beam acoustic tweezer equipped with a high-frequency array transducer could trap multiple particles both at the main lobe and at the grating lobes. In summary, there is significant evidence showing that high-frequency ultrasound microbeam non-invasively controls micron-sized particles or cells with advanced spatial resolution.

### 3.2. Cellular Application II: Acoustic Stimulation

Research into cellular responses to low-frequency ultrasound has been extensive across various cell types [39,40,41,42,43]. However, the advent of high-frequency ultrasound microbeams has greatly enhanced spatial specificity on the order of tens of micrometers. Hwang et al. [44] conducted a feasibility study on mechanical cell stimulation using a 200 MHz single-element ZnO transducer. They found that fluorescence intensity decreased more in normal cells (MCF-12F) compared to cancer cells (MDA-MB-435), with both cell types showing greater intensity reduction following ultrasound stimulation. In a subsequent study, a high-frequency ultrasound microbeam generated by a 200 MHz single-element LiNbO_3_ transducer could induce an elevation of calcium ions (Ca^2+^) in breast cancer cells, with a more pronounced effect in highly invasive MDA-MB-231 cells compared to weakly invasive MCF-7 cells [45]. Additionally, Hwang et al. [46] provided experimental evidence that a high-frequency ultrasound microbeam could induce increases in cytoplasmic Ca^2+^ in human umbilical vein endothelial cells (HUVECs) without direct contact. Meanwhile, a novel method for assessing the invasion potential of cancer cell populations was introduced, which they tested in prostate and bladder cancer cell lines using a 38 MHz single-element LiNbO_3_ transducer [47]. Their research demonstrated that ultrasound stimulation in invasive cells initiates a calcium wave that propagates from the cells at the transducer focus to neighboring cells over distances exceeding a few millimeters. Yoon et al. [48] investigated the mechanotransduction pathway involved in low-intensity focused ultrasound-induced Ca^2+^ mobilization in human mesenchymal stem cells (hMSCs) to elucidate the mechanism of action of high-frequency microbeams. Using a 47 MHz single-element LiNbO_3_ transducer, they found that connexin 43 (Cx43) hemichannels respond to the ultrasound stimulus, which results in the release of Adenosine triphosphate (ATP) into the extracellular space. This ATP subsequently elicits G-protein-coupled P2Y1 purinergic receptors on the cell membrane, triggering the production of IP3 from PLC and the subsequent release of Ca^2+^ from intracellular stores. In another study, Yoon et al. [49] demonstrated that low-intensity focused ultrasound using a 45 MHz single-element LiNbO_3_ transducer can induce distinctive oscillatory Ca^2+^ patterns in the HIT-T15 β-cell line. With ATP release from mechanosensitive hemichannels serving as the signaling mediator, the effect is mediated by the endogenous purinergic signaling pathway. Additionally, Lee et al. [50] showed that high-frequency microbeam can activate endoplasmic reticulum (ER)-localized PANX1 channels, resulting in the release of intracellular Ca^2+^. Using a 46 MHz single-element LiNbO_3_ transducer, microbeam stimulation induced the release of specific chemokines and cytokines from invasive PC-3 cancer cells. They further demonstrated that adjusting the ultrasound intensity allowed for the modulation of the profile of the cytokines and chemokines. Collectively, non-contact high-frequency ultrasound microbeams induce distinctive Ca^2+^ dynamics in various cell types in a non-invasive manner.

### 3.3. Cellular Application III: Acoustic Transfection

In contrast to previous methods that use low-frequency ultrasound with microbubble oscillations [51,52,53,54,55], Yoon et al. [56] demonstrated that high-frequency ultrasound beams can directly and remotely perturb the lipid bilayer of the cell membrane. This approach allows for the delivery of exogenous molecules into a targeted single cell with advanced spatial resolution on the order of tens of micrometers. They showed that this technique effectively increased intracellular Ca^2+^ concentrations and facilitated the delivery of propidium iodide (PI) and 3 kDa dextran labeled with Alexa 488 using highly focused 150 and 210 MHz LiNbO_3_ transducers, while simultaneously performing live cell imaging on HeLa cells. Yoon et al. [57] further demonstrated the application of high-frequency ultrasound transducers for the intracellular delivery of DNA plasmids, mRNAs, recombinant proteins, and CRISPR-Cas9 systems into HEK 293 and HeLa cells. These findings provide experimental evidence supporting the potential of the acoustic-transfection technique for precise genome editing using CRISPR-Cas9. Additionally, Kim et al. [58] optimized stimulation parameters to improve delivery efficiency and cell membrane permeability while minimizing membrane disruption across four different human cancer cell lines. They successfully employed the optimized parameter values to deliver a DNA plasmid encoding transient mCherry fluorescence into epiblast stem cells.

### 3.4. Estimation and Measurement of Acoustic Radiation Force and Trapping Force

The acoustic pressure field for high-frequency ultrasound exceeding 60 MHz cannot be precisely measured with current technology [59]. To address this limitation, Kim et al. [58] estimated the maximum pressures under various stimulation conditions at the ultrasonic focus using the commercial finite element modeling software PZFlex Professional (Cupertino, CA, USA). Their simulations showed maximum acoustic pressure values of 2 MPa, with a calculated mechanical index (MI) of 0.15 at a center frequency of 182 MHz. This MI is significantly lower than the highest MI of 1.9 set by Food and Drug Administration (FDA) regulations. Additionally, Kim et al. [60] introduced a new method for measuring the acoustic radiation force of an ultrasonic transducer with a center frequency of 130 MHz. They found that an acoustic radiation force of 42.4 μN, equivalent to 0.54 MPa at the focus, is sufficient for successful calcium transport between two cells using a FRET biosensor. In addition to acoustic radiation force estimation and measurement, Lee et al. [61] introduced a viscous drag force method to calibrate the trapping force of a 24 MHz piezo-composite focused transducer, by comparing the trapping force with known viscous drag forces. Li et al. [62] also proposed a simple acceleration-mass method to determine the effective trapping force of a 70 MHz transducer, achieving measurements in the range of a few piconewtons. To further achieve the precision measurement, Lim et al. [63] integrated a micropipette aspiration approach and measured acoustic trapping forces in the nanonewton range and trap stiffness for a 5 μm polystyrene microbead using a 110 MHz transducer by comparing with forces generated by a micropipette. They applied this method for the simultaneous trapping and measurement of single-object characteristics [64], as well as for measuring inter-red blood cell (RBC) forces of 391 pN using a 410 MHz transducer [35].

## 4. Integrating Machine Learning with High-Frequency Ultrasonic Signals

In this review, we shift the focus from the traditional use of high-frequency ultrasound images for diagnosis and analysis to a more advanced approach that leverages high-frequency signals in combination with machine learning (ML) to extract and analyze detailed cellular-level information. This integration marks an advancement in biomedical applications, particularly in the diagnosis and analysis of cancer and blood cells. The high resolution provided by high-frequency ultrasound is critical in the medical field, enabling the detailed information of cellular structures and functions. When paired with ML techniques, this technology enhances diagnostic accuracy and efficiency, paving the way for automated, precise, and objective analysis that surpasses conventional methods. Machine learning algorithms, especially convolutional neural networks (CNNs) and other deep learning models, excel at rapidly processing and analyzing vast quantities of ultrasound data, identifying patterns and anomalies that might elude human detection. The powerful synergy between high-frequency ultrasound and ML not only automates diagnostic processes but also minimizes human error and subjective interpretation, leading to more reliable and consistent outcomes.

### 4.1. Cancer Cell Analysis

In cancer cell analysis, high-frequency ultrasound captures intricate details of cell morphology and behavior. These details are crucial for assessing characteristics such as cell deformability and elasticity, which are indicators of invasiveness and metastatic potential. ML models analyze these cell images to develop accurate and efficient methods for classifying cancer cells and predicting their behavior.

**Figure 3:**
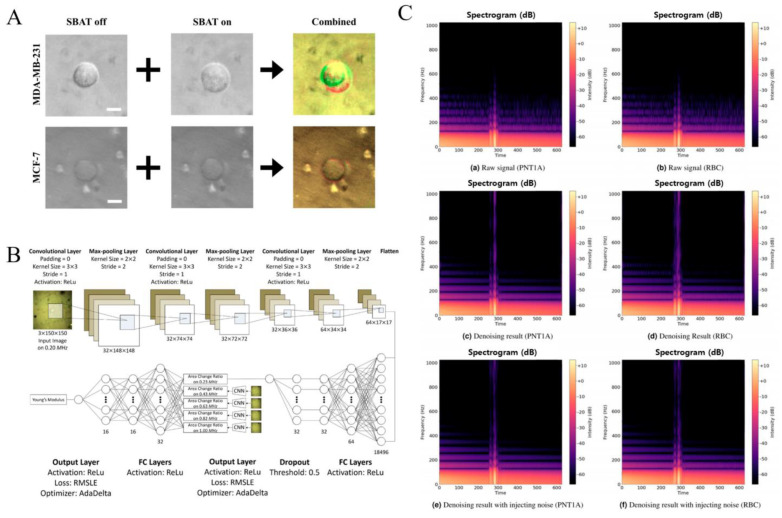
Comparative analysis of image preprocessing, proposed CNN models, and denoising autoencoder performance in cell imaging (**A**). A comparison of original and preprocessed images shows that subtle deformations in MDA–MB–231 and MCF–7 cells become clearer after preprocessing. Noise is reduced, and cell boundaries are highlighted in red and green, making the images more robust. Scale bars represent 10 µm. Reproduced with permission [65], copyright 2020, MDPI (**B**). The proposed models for measuring area change ratios and estimating non-linear elastic moduli include a CNN model for area changes and an MLP model for non-linear elastic moduli. Reproduced with permission [66], copyright 2022, Nature Portfolio. (**C**) The results of the denoising autoencoder models show spectrograms for a PNT1A cell (left) and an RBC (right). The X-axis represents time, and the Y-axis represents frequency, with color brightness indicating intensity. (**a**,**b**) show the original signals, (**c**,**d**) display results from the 1D CNN denoising autoencoder, and (**e**,**f**) show the results with added Gaussian noise. Reproduced with permission [67], copyright 2022, Nature Portfolio.

Recently, Lim et al. employed single-beam acoustic tweezers (SBATs) and a CNN to classify cancer cells based on deformability, providing objective metrics for invasiveness with precision and recall exceeding 0.95 [65]. This method is an alternative to traditional histological analysis, significantly reducing evaluation time and increasing accuracy by automating the process. The study utilized a custom-designed 50 MHz SBAT transducer to capture images of deformed human breast cancer cells, which were then analyzed using CNN-based classification methods enhanced with data augmentation techniques, as shown Figure 3A. The CNN model used to detect invasive cancer cells was a shallow model, consisting of three convolutional layers, three max-pooling layers, and two fully connected (FC) layers, and can achieve sufficient accuracy without overfitting. They used k-fold cross-validation and fluorescence cell images for validation, and the model successfully classified cells into two groups, MDA-MB-231 and MCF-7, based on the invasiveness of cells. This automated approach provides a reliable measure of the metastatic potential of cancer cells, offering a substantial improvement over subjective manual evaluations.

More recently, Lee et al. used CNNs and multilayer perceptrons (MLPs) to measure the non-linear elastic moduli of live cells, distinguishing between invasive and non-invasive breast cancer cells [66]. This method enhances accuracy and reduces the labor intensity associated with manual measurements. By obtaining photomicrographs of cells under varying pressure levels using SBAT and preprocessing these images to highlight changes in cell areas, the CNN model is trained to measure cell area changes. The CNN model for area changes includes three 2-D convolutional layers, three max-pooling layers, three fully connected layers, and one dropout layer. The activation function of the convolutional layers and fully connected layers is a rectified linear unit (ReLu), and the threshold of the dropout layer is set as 0.50. The MLP model for non-linear elastic moduli comprises three fully connected layers that consist of 32, 16, and 16 nodes, respectively, that learn the correlations between these changes and cell deformability, as shown in Figure 3B. To train the MLP model, the mean absolute error (MAE), mean squared error (MSE), root mean squared error (RMSE), mean absolute percentage error (MAPE), mean square logarithmic error (MSLE), and root mean squared logarithmic error (RMSLE) were applied as loss functions, and SGD, RMSprop, Adagrad, AdaDelta, and Adam as optimizers. This dual approach not only automates the assessment process but also provides a robust framework for distinguishing between different types of cancer cells based on their physical properties.

Jeon and co-workers developed a system integrating label-free acoustic sensing with deep learning that enables the classification of cell types by analyzing backscattered ultrasound signals [67]. This method eliminates errors associated with manual settings and time-consuming postprocessing, offering reliable and precise classification for personalized cancer medicine. The study applied a one-dimensional (1D) convolutional autoencoder to denoise signals and used data augmentation techniques to enhance robustness. The denoised backscattered signals were then classified into specific cell types using CNN models for three types of signal data representations, including 1D CNN models for waveform and frequency spectrum analysis and two-dimensional (2D) CNN models for spectrogram analysis, as shown in Figure 3C. This approach was validated by classifying different types of cells and polystyrene microspheres, demonstrating the effectiveness of the neural network models in providing accurate and efficient cell-type classification.

### 4.2. Blood Cell Analysis

**Figure 4:**
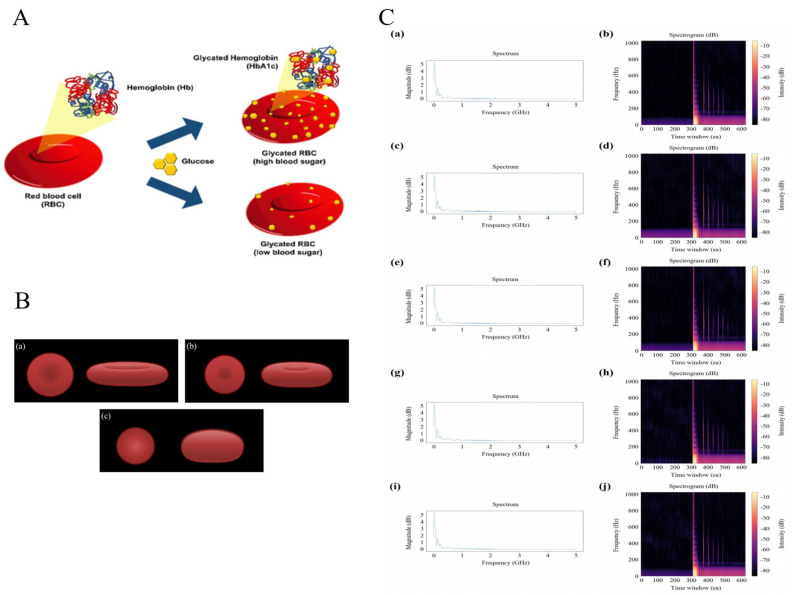
Analysis of glycated hemoglobin, sodium-induced RBC variations, and spectral patterns across disease stages (**A**). Glycated red blood cells: Glucose binds with hemoglobin in red blood cells to form glycated hemoglobin (HbA1c), with the accumulation of HbA1c varying in relation to blood glucose levels. Reproduced with permission [68], copyright 2024, Elsevier (**B**). RBC changes due to sodium concentration: (**a**) hypernatremia–decreased volume, increased diameter; (**b**) normal; (**c**) hyponatremia–increased volume, decreased diameter. Reproduced with permission [69], copyright 2024, IEEE Inc. (**C**). Spectra and spectrograms for five stages of disease classification (125~185 µmol/mL): (**a**) spectrum at 125 µmol/mL, (**b**) spectrogram at 125 µmol/mL, (**c**) spectrum at 140 µmol/mL, (**d**) spectrogram at 140 µmol/mL, (**e**) spectrum at 145 µmol/mL, (**f**) spectrogram at 145 µmol/mL, (**g**) spectrum at 155 µmol/mL, (**h**) spectrogram at 155 µmol/mL, (**i**) spectrum at 185 µmol/mL, (**j**) spectrogram at 185 µmol/mL. Concentrations at each stage were chosen based on symptom onset or the threshold between normal and abnormal. Reproduced with permission [69], copyright 2024, IEEE Inc.

High-frequency ultrasound combined with machine learning also offers non-invasive diagnostic techniques for blood cell analysis, particularly for conditions such as diabetes and dysnatremia. High-frequency ultrasound and CNNs have been used to diagnose diabetes by observing changes in red blood cells (RBCs) due to glucose concentrations [68]. This method analyzes acoustic reflection signals to detect glycated hemoglobin (HbA1c) with a high classification accuracy of 0.98, providing a non-invasive alternative to traditional blood tests. The study incubated RBCs with different glucose concentrations and collected acoustic reflection signals using a custom-designed 90 MHz transducer, as shown in Figure 4A. The CNN was then applied to the frequency spectra and spectrograms of these signals to identify correlations between changes in RBC properties due to glucose concentration and the signal features. This non-invasive diagnostic technology promises to facilitate in vivo diagnosis without the need for blood collection, improving patient comfort and diagnostic efficiency.

In addition, Nam et al. utilized single-beam acoustic microbeam (SBAM) and CNNs to measure sodium concentrations in blood, offering a reliable and rapid diagnostic tool for dysnatremia [69]. This method analyzes reflected signals from RBCs influenced by varying sodium concentrations, categorizing them into five groups—hyponatremia, normal, mild hypernatremia, moderate hypernatremia, and severe hypernatremia—and achieving high accuracy rates of 0.961 for ten-level classifications and 0.942 for five-level classifications, as shown in Figure 4B. The technology involves fabricating a 90 MHz transducer to obtain reflected signals from RBCs, which are then analyzed using CNNs to classify the blood samples based on the sodium chloride (NaCl) concentration. Figure 4C presents the spectra and spectrograms corresponding to five distinct disease stages, with concentrations chosen based on either symptom onset or the normal-abnormal threshold. The concentrations are as follows: (a, b) 125 µmol/mL, (c, d) 140 µmol/mL, (e, f) 145 µmol/mL, (g, h) 155 µmol/mL, and (i, j) 185 µmol/mL. The models in this research were divided into two main parts: a feature extractor and a classifier. The convolutional block, comprising convolution layers, max-pooling layers, and dropout layers, was responsible for extracting key cell characteristics from reflection signals. A 1-D convolutional layer was applied to analyze the frequency spectra, while a 2-D convolutional layer was used for processing spectrograms of the reflection signals. The dense block then classified each signal based on sodium concentration levels. The study demonstrated the potential of SBAM and CNN technologies for the efficient quantification of sodium concentration in blood, providing a non-invasive method for diagnosing dysnatremia. This approach not only reduces analysis time but also offers higher reliability and accuracy compared to traditional blood sodium tests, which can be influenced by various factors such as inspection equipment and methods.

## 5. Limitations and Potential Applications

High-frequency ultrasound inherently has limited penetration depth, restricting its use on superficial regions [70,71,72,73,74]. However, this trade-off allows for significantly enhanced spatial resolution, enabling the visualization of detailed superficial structures and manipulation of cellular activities at the single-cell level [75,76,77,78,79]. Therefore, understanding the cellular mechanical interactions of ultrasound beams is crucial for translating these findings to in vivo studies in both animals and humans. These technological advancements pave the way for improvements in personalized medicine, allowing for more accurate and timely diagnoses, reducing the need for invasive procedures, and improving patient experiences. As research progresses, the potential for high-frequency ultrasound and machine learning to revolutionize medical diagnostics and treatment becomes increasingly evident, with promising applications extending beyond cancer and blood cell analysis. This synergy is set to drive significant progress in healthcare, leading to better health outcomes and a higher standard of care.

## 6. Conclusions

In conclusion, high-frequency ultrasound represents a significant advancement in imaging, cellular function control, and the integration of machine learning. High-frequency ultrasound imaging systems have transitioned from mechanical scanners to array-based systems that support various imaging modes, such as B-mode and color Doppler, thereby significantly contributing to pre-clinical research. The introduction of portable high-frequency ultrasound systems is anticipated to enhance their application in clinical settings. These systems leverage high-frequency ultrasound microbeams for the non-invasive manipulation of micron-sized subjects through methods like acoustic tweezers and stimulation, achieving notable spatial resolution. Moreover, the integration of machine learning with high-frequency ultrasound is transforming medical diagnostics, offering new opportunities for early disease detection, tailored treatment plans, and enhanced patient outcomes.
